# Structure and Physiological Actions of Ghrelin

**DOI:** 10.1155/2013/518909

**Published:** 2013-11-28

**Authors:** Christine Delporte

**Affiliations:** Laboratory of Pathophysiological and Nutritional Biochemistry, Université Libre de Bruxelles, 808 Route de Lennik, Bat G/E-CP611, 1070 Brussels, Belgium

## Abstract

Ghrelin is a gastric peptide hormone, discovered as being the endogenous ligand of growth hormone secretagogue receptor. Ghrelin is a 28 amino acid peptide presenting a unique *n*-octanoylation modification on its serine in position 3, catalyzed by ghrelin *O*-acyl transferase. Ghrelin is mainly produced by a subset of stomach cells and also by the hypothalamus, the pituitary, and other tissues. Transcriptional, translational, and posttranslational processes generate ghrelin and ghrelin-related peptides. Homo- and heterodimers of growth hormone secretagogue receptor, and as yet unidentified receptors, are assumed to mediate the biological effects of acyl ghrelin and desacyl ghrelin, respectively. Ghrelin exerts wide physiological actions throughout the body, including growth hormone secretion, appetite and food intake, gastric secretion and gastrointestinal motility, glucose homeostasis, cardiovascular functions, anti-inflammatory functions, reproductive functions, and bone formation. This review focuses on presenting the current understanding of ghrelin and growth hormone secretagogue receptor biology, as well as the main physiological effects of ghrelin.

## 1. Introduction

Ghrelin is a unique 28 amino acid peptide containing an *n*-octanoyl group on the serine in position 3 that was purified from rat stomach in 1999 [1, 2]. Ghrelin is the only known peptide hormone modified by a fatty acid. Ghrelin is synthesized by the endocrine X/A-like cells of the fundus mucosa representing about 20% of gastric mucosal cells in humans [3–5]. Ghrelin is the natural ligand for the growth hormone secretagogue (GHS) receptor (GHS-R) cloned in 1996 [6]. Circulating ghrelin consists of more than 90% of desacyl ghrelin and less than 10% acyl ghrelin [7]. However, the acyl group of ghrelin is essential for its binding to GHS-R and the concomitant activation of the inositol triphosphates/calcium pathway [1, 2].

In addition to the stomach [1, 8], ghrelin is expressed in many tissues such as duodenum, jejunum, ileum, colon, lung, heart, pancreas, kidney, testis, pituitary, and hypothalamus [3, 9–13].

The major biological functions of ghrelin include the secretion of growth hormone, the stimulation of appetite and food intake, the modulation of gastric acid secretion and motility, and the modulation of the endocrine and exocrine pancreatic secretions.

## 2. From Ghrelin Gene to Ghrelin-Related Peptides

### 2.1. Transcriptional and Translational Mechanisms

**Figure 1:**
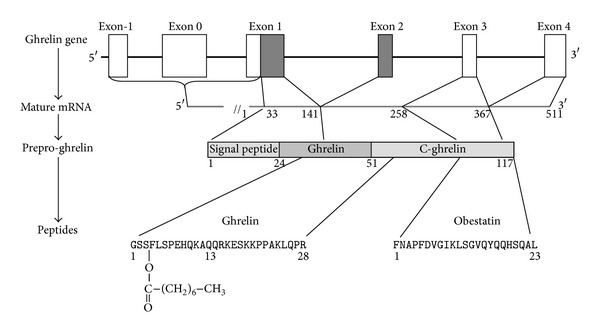
From ghrelin gene to ghrelin peptide. Following the transcription of ghrelin gene containing 2 exons (black boxes) and 5 introns (white boxes), mature ghrelin mRNA is processed into preproghrelin and finally into several peptides, namely, ghrelin and obestatin.

The human ghrelin gene is located on the short arm of chromosome 3 (3p25-26) and contains six exons (2 are noncoding) and 4 introns and encodes a 511 bp mRNA [14–16] (Figure 1). Preproghrelin (117 AA) contains 23 AA signal peptide and a 94 AA segment corresponding to proghrelin [17] (Figure 1). Proghrelin is made of the 28 AA ghrelin peptide and a 66 AA carboxyterminal peptide called C-ghrelin [16, 18, 19] (Figure 1). C-ghrelin is further processed to a 23 AA peptide named obestatin [20] (Figure 1). Besides, alternative splicing of the human ghrelin gene generates additional transcripts coding for other peptides including des-Gln14-ghrelin [16, 21] (Figure 1). Finally, antisense transcripts (transcribed from the antisense strand), unlikely coding for proteins, may produce noncoding RNAs, possibly involved in posttranscriptional and/or posttranslational gene regulation [16].

The enzymes responsible for processing preproghrelin into ghrelin include signal peptidase cleaving at Arg23, prohormone convertase 1/3 (PC 1/3) cleaving at Arg51 (generating ghrelin 1–28) [22], and carboxypeptidase-B like enzyme cleaving at Pro50 (generating ghrelin 1–27) [23] (Figure 1). The involvement of prohormone convertase 2 (PC2) and furin in the preproghrelin processing remains controversial [22, 24, 25]. The enzymes responsible for processing preproghrelin into obestatin remain yet to be identified.

The data published so far on the human ghrelin gene structure strongly suggests a higher level of complexity than previously recognized. Additional studies will be required to address the fine-tuning regulatory mechanisms of the ghrelin gene transcription and processing that may depend upon tissue type and physiological conditions. Identification and characterization of novel transcripts as well as novel derived-peptides from the human ghrelin gene would raise challenging questions concerning their physiological and pathophysiological implications.

### 2.2. Posttranslational Acylation Mechanism

Nascent ghrelin peptides, derived from the human ghrelin gene transcription and translation, are subjected to a unique posttranslational modification consisting in the acylation of the hydroxyl group of the Ser3 [1]. Both ghrelin 1–28 and ghrelin 1–27 are subjected to acylation, essentially by an octanoyl group (C8:0) and more rarely by a decanoyl (C10:0) or decanoyl (C10:1) group [23]. Ingestion of either medium-chain fatty acids or medium-chain triglycerides increases ghrelin acylation [26, 27]. The enzyme responsible for ghrelin acylation is ghrelin *O*-acyl transferase (GOAT) belonging to the family of membrane *O*-acyl transferases (MBOAT) [28, 29]. Ghrelin and GOAT colocalize in gastric X/A like cells [28]. Diets enriched with C8 medium-chain or C10 medium-chain triglycerides led to a modification in the proportions of octanoyl or decanoyl ghrelin stored in the same granules in gastric X/A like cells, suggesting that GOAT is likely to use the most available substrate to perform ghrelin acylation [30]. Ghrelin acylation is believed to occur in the endoplasmic reticulum prior to the processing of proghrelin by various proteases on either the preproghrelin and/or the proghrelin precursors [22, 29, 31, 32]. GOAT acylates ghrelin with fatty acids ranging from C:7 to C:12 [28]. Presumed donors of acyl group are acyl-CoA, even though it remains unclear how acyl-CoA could get into the endoplasmic reticulum lumen [29]. In an *in vitro *assay of GOAT activity, GOAT was shown to possess a preference for *n*-hexanoyl-CoA over *n*-octanoyl-CoA as an acyl donor [32]. However, several lines of evidence suggest that the intracellular concentrations of *n*-acyl-CoA are likely to affect GOAT substrate specificity. Mutation in position 338 of GOAT, at the MBOAT-conserved histidine residue, abolishes ghrelin octanoylation [28].

The investigation of GOAT substrate specificity, using an *in vitro* assay for GOAT activity, indicates that Gly1, Ser3, and Phe4 surrounding Ser3 in proghrelin represent crucial amino acids for GOAT activity [33]. Pentapeptides corresponding to the first five N-terminal amino acids of ghrelin with its C-terminal end amidated and with an ester or amide (conferring a better efficiency than the ester) linkage between Ser3 and C:8 competitively inhibit GOAT activity [34]. Similar pentapeptide with Ala3 instead of Ser3 represents efficient GOAT inhibitor that can no longer be used as a substrate [34]. A peptide-based bisubstrate analog GO-CoA-Tat, a potent inhibitor of GOAT, improves glucose tolerance and reduces weight gain in wild-type mice fed with medium-chain triglycerides diet, but not in ghrelin-knockout mice [35]. Furthermore, GO-CoA-Tat decreased circulating octanoyl ghrelin levels and body weight in mice fed with high fat diet [36]. Very recently, mutations and chemical modifications of a novel fluorescent substrate peptide for GOAT allowed defining specific interactions without GOAT active site playing a role in ghrelin recognition [37]. These recent finding should allow developing more potent and specific GOAT inhibitors for evaluating the GOAT-induced ghrelin acylation pathway as a new therapeutic target.

While rat gastric GOAT mRNA levels are similar in fed and 48 h-fasted animals, they increased in response to leptin administration in fasted animals, indicating that GOAT is a leptin-regulated gene [38]. Higher GOAT mRNA levels detected in chronic restricted-nutritional conditions, such as anorexia nervosa, could account for the higher acyl ghrelin levels measured [39].

GOAT knockout mice are characterized, as expected, by a total absence of acylated ghrelin [40].

In agreement with its physiological functions, ghrelin acylation, via GOAT, is involved in eating behavior. In homeostatic eating (when food intake is driven by necessity, due to energy deficiency as perceived by the brain and body), GOAT knockout mice models can exhibit similar or contrasting phenotypes depending on the type of diet. GOAT knockout mice fed with standard chow diet demonstrated higher desacyl ghrelin than their wild-type littermates but had similar body weight, fat mass, and food intake [40, 41]. However, GOAT knockout mice fed with high fat diet displayed either similar body weight, body composition, and food intake to their wild-type littermates [41] or lower body weight, no changes in body composition, and increasing food intake [40]. GOAT knockout mice fed with high fat diet rich in medium-chain triglycerides displayed lower body weight and fat mass, despite increase food intake [40]. These discrepant phenotypes observed could result from the distinct genetic background of the mice models used. In hedonic feeding (when food intake is motivated primarily by pleasure), GOAT knockout mice phenotypes suggested that GOAT is a critical modulator in food reward. Indeed, GOAT knockout mice displayed an attenuated food motivation in an operant responding model when deprived of food for 24 h [42] and a decreased hedonic feeding response in a “dessert effect” protocol [42].

GOAT knockout in leptin-deficient ob/ob mice does not improve glucose tolerance or body adiposity, suggesting that the desacyl/acyl ghrelin ratio has no major effects on glucose homeostasis in a model of massive obesity and glucose intolerance [43].

Very recently GOAT has been detected in human plasma and its expression level was positively correlated with body mass index and negatively correlated with ghrelin level when evaluating normal subjects and subjects with either anorexia nervosa or obesity [44]. It has to be noted that in that particular study ghrelin level was the same in obese patients and normal weight subjects [44], as opposed to other previous studies showing either decreased ghrelin levels [39, 45] or increased ghrelin levels [46, 47]. Further studies using large cohorts of subjects will be required to assess if GOAT indeed counteracts the effects of ghrelin and contributes to the development or maintenance of anorexia nervosa and obesity.

Knocking out GOAT, possibly in combination with deficiency in ghrelin and/or GHSR, should allow assessing the physiological consequences of a deficiency in ghrelin acylation and/or ghrelin signaling. Moreover, GOAT represents a useful pharmacological target in the treatment of obesity and other diseases [48]. Finally, further studies should contribute to a better understanding of the role of GOAT in the control of ghrelin acylation and its subsequent effects.

Ghrelin and desacyl ghrelin can undergo another posttranslational modification: phosphorylation on Ser18 by protein kinase C [49]. Compared to nonphosphorylated ghrelin and desacyl ghrelin, both phosphorylated forms exhibited lower binding capacity to phosphatidylcholine : phosphoserine sucrose loaded vesicles [49]. However, additional studies are required to determine if ghrelin phosphorylation can occur in cells under specific conditions and if so what would be the impact of such phosphorylation on the subcellular localization and biological function of the peptide.

## 3. Ghrelin-Related Peptides

### 3.1. Sequence Homologies between Mammalian Ghrelin

**Figure 2:**
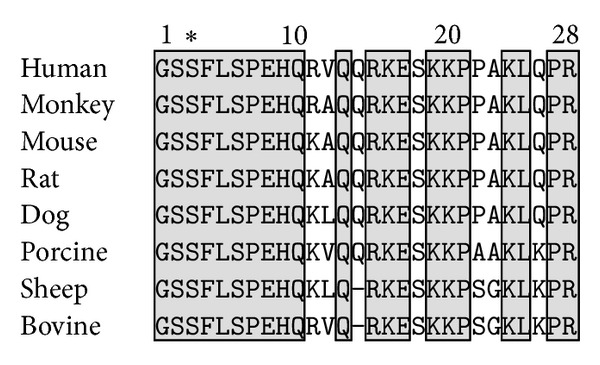
Sequence homologies between mammalian ghrelin. Highlighted boxes indicate amino acid homologies among ghrelin from mammalian species. The acylation of ghrelin on the third conserved serine is indicated by a∗.

Ghrelin has been purified from various mammalian species such as human [1], rat [1], and mouse [50] (Figure 2). All mammalian ghrelin sequences present a strict sequence conservation of the first 10 N-terminal AA and the acylated-Ser3. Human and rat ghrelin are identical with the exception of AA in positions 11 and 12. Ovine and bovine ghrelin sequences are made of 27 AA, rather than 28, and are lacking Gln14.

### 3.2. Structure-Activity Relationships of Ghrelin-Related Peptides

**Figure 3:**
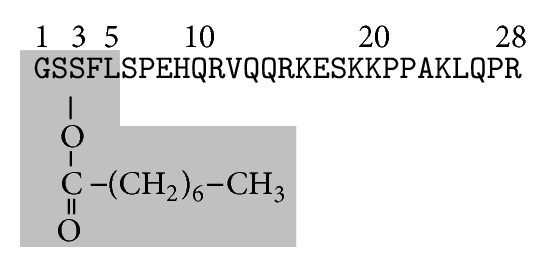
Minimal active core sequence of ghrelin required for GHS-R1A activation. The highlighted box indicates the minimal active N-terminal pentapeptide core of ghrelin sequence required for GHS-R1A activation.

Several studies have investigated the structure-activity relationship of ghrelin peptides. The presence of acyl group on Ser3 is required for most of the observed biological activities of ghrelin [51, 52]. The position of the octanoylated Ser is important as C8:0 Ser2 conserved the activity of the peptide while C8:0 Ser6 or C8:0 Ser18 decreased it [53]. Maximal ghrelin activity, conferred by C8:0 Ser3, is maintained by C10:0 Ser3, C12:0 Ser3, and C16:0 Ser3 but drastically decreased by C4:0 Ser3 or C2:0 Ser3 [51]. While the replacement of Ser3 by Trp3 maintains the activity of ghrelin, its replacement by aliphatic AA (such as Val, Leu, or Ile) completely inhibits its activity [51]. The ester bound between C8:0 and Ser3 is not indispensable for ghrelin activity as it can be replaced, without affecting the activity, by thioether or ether bounds [51]. The N-terminal positive charge and Phe4 are necessary for ghrelin activity and recognition by GHS-R1A [54]. Systematic C-terminal truncation of ghrelin identified the N-terminal pentapeptide of ghrelin, including C8:0 Ser3, to be the minimal peptide fragment equipotent to ghrelin [52, 55]. In addition, amidation of the C-terminus increased the activity of the ghrelin fragments [52, 55], while N-acetylation drastically decreased it [54, 55]. Bound to lipids, both acyl and desacyl ghrelin adopt a short *α*-helix conformation, respectively, from Pro7 to Glu13 and Pro7 to Glu14, surrounded by more flexible N- and C-termini [56, 57]. The minimal active core sequence of ghrelin required for GHS-R1a activation is summarized in Figure 3.

### 3.3. Circulating Ghrelin

In human, circulating ghrelin consists of desacyl ghrelin (>90%), acyl ghrelin, and C-ghrelin [7, 18, 58]. Circulating C-ghrelin is decreased by about 80% in rat [59] and in humans [8, 60] following surgical gastric mucosa removal. It presently remains unknown if ghrelin and desacyl ghrelin are both secreted into the bloodstream via similar or distinct secretory pathway(s). In rats, gastric ghrelin is degraded by deacylation and N-terminal proteolysis [61], and deacylation is performed by lysophospholipase I [62]. The high desacyl/acyl ghrelin ratio in the circulation can be explained by the shorter half life of ghrelin compared to desacyl ghrelin [63] and plasma ghrelin deacylation [58, 61]. Butyrylcholinesterase and other esterase(s), such as platelet-activating factor acetylhydrolase, are responsible for ghrelin deacylation in human serum, while carboxylesterase accounts for ghrelin deacylation in rat serum [61, 64]. Interestingly, butyrylcholinesterase knockout mice fed with standard chow diet (5% fat) displayed normal body weight, while those fed with high fat diet (11% fat) became obese [65]. As increased ghrelin levels cannot explain obesity, butyrylcholinesterase could play a role in fat utilization [65]. Paraoxonase was also suggested to participate in ghrelin deacylation in human serum [66]. However, this hypothesis was refuted as EDTA had no effect on ghrelin deacylation and there was a negative correlation between desacyl ghrelin levels and paraoxonase activity [61]. Desacyl ghrelin mostly circulates as a free peptide, while acyl ghrelin circulates bound to lipoproteins [64, 66]. The acyl group is required for ghrelin interaction with triglyceride-rich lipoproteins and low-density lipoprotein, while N- and C-terminal ends of ghrelin are required for its binding to high-density lipoproteins and very high-density lipoproteins. It is therefore hypothesized that triglyceride-rich lipoproteins mostly transport acyl ghrelin, while high-density and very high-density lipoproteins transport both acyl and desacyl ghrelin [64].

Due to the rapid degradation of ghrelin into the circulation, it is necessary to collect blood samples under appropriate optimal conditions warranting the intact conservation of the peptide. Several studies have evaluated the effect of blood collection and storage conditions on the measurement of ghrelin levels [67–71]. To ensure ghrelin stability, it is now strongly recommended to collect blood samples with EDTA-aprotinin (or other proteases inhibitors) under cooled conditions and proceed to the sample acidification and dilution prior to ghrelin measurement [69, 70]. Despite the obvious involvement of esterases in ghrelin degradation, the use of esterases inhibitors has not been recommended during blood sample collection. This is quite surprising considering that the addition of PMSF (an inhibitor of serine esterase) and eserine salicylate (an inhibitor of butyrylcholinesterase) has been shown to increase the recovery of acyl ghrelin in blood samples [64]. Additional studies should be performed to assess the usefulness of combining esterases inhibitors to the recommended guidelines to further improve ghrelin stability.

A very recent study attempted to evaluate the pharmacokinetic parameters of infused acyl ghrelin, desacyl ghrelin, or both combined in healthy human subjects with normal liver and kidney functions [72]. Following acyl ghrelin infusion, mean half life of acyl ghrelin was in the range of 9–11 min, while that of total ghrelin (acyl + desacyl ghrelin) was in the range of 35 min [72], in agreement with previous studies [63, 73, 74]. The clearance time of acyl ghrelin was about three times higher than the one for acyl ghrelin [72]. Data from this study revealed that acyl and desacyl ghrelin have different metabolic rates in the circulation with different rates of clearance [72]. Infusion of desacyl ghrelin increased desacyl concentrations without affecting acyl ghrelin levels [72, 74]. Combined acyl and desacyl infusion led to increased acyl and desacyl ghrelin concentrations, to the same extent as that observed with individual infusion [72]. Moreover, the data indicated that acyl ghrelin was actively deacylated in the plasma [72]. The relatively constant acyl ghrelin/desacyl ghrelin ratio, measured at baseline and during infusion, suggests that both desacyl ghrelin production (due to acyl ghrelin deacylation) and desacyl ghrelin elimination were increased in similar proportions [72]. Determination of pharmacokinetic parameters of acyl ghrelin and total ghrelin is of prime importance for conducting appropriate clinical research. Furthermore, the modification of pharmacokinetic parameters in diseased subjects is likely to influence clinical research data and conclusions.

Other important considerations should be taken into account for proper ghrelin level assessment such as the nutritional status of the individuals, the time point at which blood samples are collected [75], and the efficiency of peptide extraction as well as the limitations of the methods used to assay ghrelin levels (acyl, desacyl, and/or total ghrelin) [76–79].

**Figure 4:**
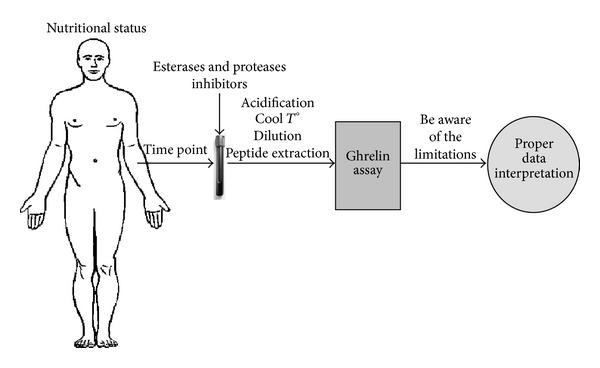
Proposed controlled steps necessary to allow accurate ghrelin levels determination. *T*°: temperature.

In the light of the rapid degradation of ghrelin in the circulation, investigators need to pay greater attention to the conditions of collection and conservation of blood samples to ensure optimal ghrelin stability. Furthermore, the nutritional status of individuals and the time point of blood sample collection should also be carefully controlled. Finally, pharmacokinetic parameters of ghrelin should be taken into account when designing clinical research. Ruling out these important issues would definitively impact the accuracy of acyl ghrelin levels determination and, consequently, and most importantly, on the assessment of its physiological and pathophysiological roles. Furthermore, a large number of studies evaluating ghrelin levels do not distinguish between total ghrelin, acyl ghrelin, and desacyl ghrelin levels. Investigators should therefore choose the best available method for ghrelin levels determination and be aware and discuss the limitations of the method used. All together, these elements are likely to contribute to the possible contradictory data published in the literature concerning the physiological and pathophysiological role of ghrelin. In conclusion, only combined controlled steps will ensure accurate ghrelin level determination and proper interpretation under physiological and pathophysiological conditions (Figure 4).

### 3.4. Tissue Distribution of Ghrelin

Ghrelin is predominantly expressed in the digestive system, with highest expression levels in the gastric mucosa [1, 8]. Gastric mucosa is composed of five endocrine cell types: enterochromaffin cells (EC), enterochromaffin-like cells (ECL), D cells, G cells, and X/A-like cells which, respectively, secrete serotonin, histamine, somatostatin, gastrin, GABA, and ghrelin. Human versus rat gastric mucosa are, respectively, composed of 30%/60–70% ECL cells, 20%/20% X/A-like cells, 22%/2.5% D cells, and 7%/0–2% of EC and G cells [5, 80]. X/A-like cells secreting ghrelin are ovoid cells located into the gastric fundus [3, 4, 81]. In the gastrointestinal tract, ghrelin expression gradually decreases from the duodenum to the colon [3, 9, 10]. Circulating ghrelin originates in vast majority from gastric mucosa and gastrointestinal tract [8, 82]. In pancreas, ghrelin immunoreactivity colocalized with glucagon-producing cells (*α* cells) [83, 84], insulin-producing cells (*β* cells) [85], a new type of endocrine cell called *ε* cells [86–88], and acinar cells [89].

Ghrelin is expressed in low amounts in the central nervous system [9]. Neurons from the arcuate nucleus of the hypothalamus, a region involved in the control of food intake, express ghrelin [1, 90]. Pituitary also contains ghrelin [11, 91].

Furthermore, ghrelin is present in other tissues such as kidneys, adrenal glands, thyroid, breast, ovary, placenta, testis, prostate, liver, gallbladder, lung, skeletal muscles, myocardium, skin, and bone [12, 13].

## 4. Ghrelin Receptors

### 4.1. GHS-R Gene and GHS-R Protein Isoforms

The ghrelin receptor, termed GHS-R, belongs to G-protein coupled receptors (GPCR) superfamily, characterized by seven transmembrane spanning helix domains [6]. Human GHS-R gene is located on chromosome 3 (3q26.2) and composed of 2 exons and 1 intron. Exon 1 codes for the GHS-R region from the extracellular N-terminal end to the 5th transmembrane helix, while exon 2 codes for the rest of the receptor [92]. Two spliced variants of GHS-R have been identified: GHS-R1A and GHS-R1B. GHS-R1A is a 366 AA protein containing 7 transmembrane helix domains, while GHS-R1B is a 289 AA protein containing only 5 transmembrane helix domains [92]. GHS-R1A and GHS-R1B possess 100% sequence homology in the nucleotide sequence coding for the first 265 AA, then the nucleotide sequence of GHS-R1B is distinct from GHS-R1A as it codes for only 24 additional AA due to the presence of a stop codon [92].

GHS-R1A belongs to the class A G-protein coupled receptors subfamily but is not closely related to the other known members. Nevertheless, GHS-R1A is often included in the class A receptors subfamily for small polypeptides comprising the motilin receptor (52% homology), neurotensin-1 and neurotensin-2 receptors (±35% homology), neuromedin-1 and neuromedin-2 receptors (±30% homology), and GPR39 (±30% homology) [92, 93].

### 4.2. GHS-R1A Signaling

Coupling of GHS-R1A to G-protein involves the 3rd intracellular loop. The lack of a 3rd intracellular loop in GHS-R1B prohibits it from coupling to G-proteins. GHS-R1A activation leads to the subsequent activation of phospholipase C, inositols triphosphates, and intracellular calcium pathways [94]. At physiological concentrations, only acyl ghrelin binds to GHS-R1A, while at supraphysiological concentrations (1 *μ*M) desacyl ghrelin appears to bind to the receptor as well [95, 96]. The cell membrane has been suggested to act as “catalyst” for ghrelin binding to its receptor. Indeed, acyl ghrelin and desacyl ghrelin are electrostatically attracted to membranes by their basic residues, but acyl ghrelin penetrates deeper due to its acyl group [96]. The acyl group of ghrelin is assumed to favor ghrelin partitioning into the lipids to increase the local concentration of ghrelin in the vicinity of the receptor, to bring ghrelin to the membrane where its binding pocket is present, and to optimize the conformation of ghrelin for improving its docking to GHS-R1A [96]. Noteworthy is that GHS-R1A possesses constitutive level activity [97] that appears to be conferred by the presence of 3 aromatic AA located in the 6th and 7th transmembrane helix domains. The proposed model supposes that these AA ensure proper docking of the extracellular end of the 7th transmembrane helix domain into the 6th transmembrane helix domain, thereby mimicking agonist activation and stabilizing the receptor in its active conformation [97]. The high basal signaling of GHS-R1A has also been demonstrated *in vivo* in the hypothalamus [98].

Several studies have been designed to develop GHS-R1A peptide and nonpeptide agonists, inverse agonists, and antagonists and to map the site of interactions of these molecules with GHS-R1A using mutational, molecular modeling and computational analyses. Despite the necessity to understand the molecular bases leading to the development of drugs modulating ghrelin signaling, the studies addressing these issues will not be detailed here as they are extremely too focused in light of the scope of this review.

The monomeric existence and functioning of GHS-R1A have been established [99]. However, growing evidence now supports the notion that GHS-R1A, similarly to other GPCRs [100, 101], may form dimers [102–116]. GHS-R1A can dimerize into homo- or heterodimers and therefore potentially affect downstream signaling. GHS-R1A was shown to function as homodimer [103, 107].

But, it has now been also shown that GHS-R1A forms heterodimers with members of the prostanoid receptor family such as vasodilatator prostacyclin receptor (IP), the vasoconstrictor prostaglandin E2 receptor subtype EP3-I (EP3-I), and thromboxane A2 (TP*α*) [108]. The consequences of such heterodimer formation include decreased GHS-R1A expression, increased intracellular GHS-R1A localization, and decreased constitutive GHS-R1A activity [108]. Though, another consequence of receptor heterodimerization may be ligand specificity switching, this has not be explored or shown for GHS-R1A.

GHS-R1A interacts with the somatostatin receptor 5 (SST5), leading to a coupling to G*α*i/o instead of G*α*q11, allowing ghrelin (rather than somatostatin) to suppress glucose-stimulated insulin secretion [113]. Moreover, the high ratio of ghrelin to somatostatin affects the formation of GHS-R1A/SST5 heterodimers [113].

The formation of heterodimers between GHS-R1A and melanocortin 3 receptor leads to a mutual signaling interference and consequently to alteration in the homeostatic control of food intake and energy balance [110, 111].

The formation between GHS-R1A and dopamine D1 (D1) and D2 (D2) receptors impacts the role of ghrelin in the regulation of rewarding and motivational eating behavior [104, 112, 114]. The formation of GHS-R1A/D1 heterodimers leads to the attenuation of GHS-R1A signaling, suggesting a switch in GHS-R1A coupling from G*α*q11 to G*α*s [104]. GHS-R1A/D2 heterodimers alter the G*α*i/o-induced signaling of D2 by inducing calcium mobilization upon dopamine stimulation that was independent of GHS-R1A-G*α*q11 mediated signaling or GHS-R1A constitutive activity [112]. Furthermore, GHS-R1A/D2 heterodimers attenuates food intake [112].

GHS-R1A forms heterodimers with serotonin 2C receptor (5-HT_2C_) and leads to the attenuation of ghrelin-induced calcium signaling [115]. GHS-R1A/5-HT_2C_ heterodimers induce the attenuation of ghrelin's orexigenic effects [116]. These data are in favor of a role of GHS-R1A/5-HT_2C_ heterodimers in homeostatic appetite signaling.

Heterodimerization definitively represents a novel mechanism for fine-tuning of GHS-R1A-mediated signaling, and introduced another level of regulatory complexity. However, the physiological relevance of such mechanism remains to be further investigated. Nevertheless, GHS-R1A dimerization with other receptors offers novel pharmacological targets and therapeutic perspectives.

### 4.3. GHS-R1B Signaling

GHS-R1B is unable to bind acyl or desacyl ghrelin [6]. GHS-R1B, considered in the past to be functionally inactive, is now believed to act as an important modulator in ghrelin-induced GHS-R1A signaling. Indeed, GHS-R1B is able to heterodimerize with GHS-R1A and to decrease the constitutive activity of GHS-R1A [102, 106, 107, 109]. GHS-R1B exerts a dominant negative effect via a conformational restriction of the GHS-R1A that becomes unable to subsequently activate G protein and recruit *β*-arrestin [117]. Furthermore, GHS-R1B can form heterodimers with neurotensin receptor 1 (NTS1) and function as a receptor for neuromedin U, thereby affecting the growth of lung cancer cells through the transactivation of its downstream signals [105].

Further studies are needed to further investigate the physiological role of GHS-R1B.

### 4.4. Desacyl Ghrelin Receptors

Despite its lack of binding to GHS-R1A at physiological concentrations, desacyl ghrelin has been shown to have numerous biological effects, suggesting that it may act via a yet unidentified receptor. Desacyl ghrelin could modulate food intake as in [118] even thought GOAT knockout mice displayed suppressed fat mass despite increase in desacyl ghrelin levels [40]. In addition, desacyl ghrelin modulates cell proliferation [76, 119–123], cell apoptosis [124–127], cell metabolism [126, 128, 129], glucose homeostasis [130–132], and body temperature [133].

Consequently, further investigations will be required to identify potential desacyl ghrelin receptor(s). The identification of desacyl ghrelin receptors would lead to a better understanding of the mechanisms and sites of action of this peptide.

## 5. Physiological Functions 

**Figure 5:**
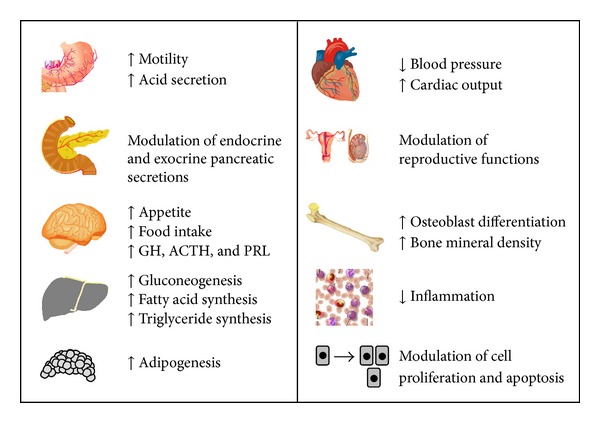
Main physiological functions of ghrelin.

Figure 5 summarizes the main physiological functions of ghrelin.

It is necessary to keep in mind that future studies are required to determine if homo- or hetero-GHS-R1A dimers are involved in the physiological actions of acyl ghrelin. Furthermore, for the desacyl ghrelin-mediated physiological effects, further studies will be necessary to identify the as yet unidentified receptor(s) involved. Finally, as it is possible that the acyl ghrelin/desacyl ghrelin ratio modulates several physiological effects, studies should be performed to analyze the impact of such parameters on the investigated biological function.

### 5.1. Growth Hormone Secretion

Via its binding to GHS-R1A, present on pituitary somatotropic cells, ghrelin is a potent stimulator of growth hormone (GH) secretion [134–137]. GH secretion is induced by both ghrelin-induced cyclic GMP/nitric oxide signaling pathway [138]. Hypothalamus also appears to be involved in the ghrelin-induced GH secretion [139]. Vagus nerve is also required for maximal ghrelin-induced GH secretion [140, 141]. Besides, ghrelin acts in synergy with GH-releasing hormone (GHRH) on GH secretion [135, 142]. However, clinical studies have led to contradictory data concerning the relationship between GH and ghrelin circulating levels [143–148]. Most of these studies measured total ghrelin levels rather than acyl ghrelin levels and did not necessarily design protocols taken into account the pulsatility of both ghrelin and GH secretion. Natural mutation in GHS-R1A resulting in loss of constitutive activity lead to impaired GH release and short stature [149, 150]. Therefore, while ghrelin is capable of stimulating GH release, its physiological involvement in GH release remains a subject of controversy. Additional studies might therefore still be valuable to further study the physiological involvement of ghrelin in GH release. Nevertheless, the potential beneficial effects of ghrelin analogs for the treatment of GH-deficiency disorders have been investigated [151].

Desacyl ghrelin is also able to induce GH secretion, possibly by modulating the GH-insulin growth factor axis [152].

Ghrelin is also able to stimulate the pituitary secretion of adrenocorticotropic hormone (ACTH), cortisol, and prolactin (PRL) [153].

### 5.2. Appetite and Food Intake

Both central and peripheral administration of ghrelin to rats induces food intake stimulation and energy expenditure reduction accounting for body weight increase [154–158]. Ghrelin administrated intravenously to human also leads to appetite increase and food intake stimulation [159].

Ghrelin is secreted in a pulsated manner as its level increases before the onset of meal, during fasting, and decreases after feeding [160, 161]. This pulsatile secretion of ghrelin suggested that ghrelin may act as a signal for meal initiation. However, it appears that peaks of ghrelin concentrations are related to meal patterns and may rise in anticipation of eating rather than elicit feeding [162].

**Figure 6:**
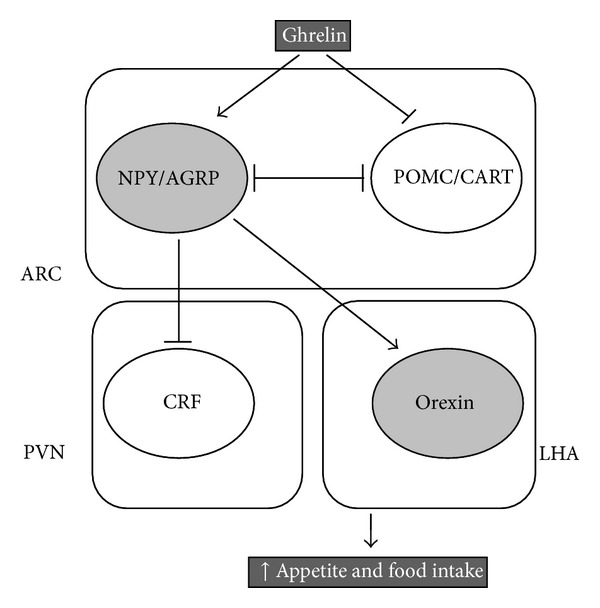
Main hypothalamic pathways involved in ghrelin-induced food intake. Arrows and lines indicate stimulation and inhibition, respectively. AGRP: agouti-related protein; ARC: arcuate nucleus; CART: cocaine- and amphetamine-regulated transcript; CRF: corticotrophin-releasing factor; LHA: lateral hypothalamic area; NPY: neuropeptide Y; POMC: proopiomelanocortin; and PVN: paraventricular nucleus.

Orexigenic and anorexigenic peptides control appetite. Among orexigenic peptides (neuropeptides Y (NPY), agouti-related peptide (AGRP), orexins, melanin-concentrating hormone (MCH), and galanin), ghrelin is the only one acting peripherally to stimulate appetite, while all other orexigenic peptides are acting centrally. Among anorexigenic peptides, some are synthesized by the hypothalamus (melanocortin (*α*-MSH), cocaine- and amphetamine- regulated transcript (CART), and corticotrophin-releasing hormone (CRH)), endocrine cells from the gastrointestinal tract (cholecystokinin (CCK), gastrin-related peptide (GRP), glucagon-like peptides 1 and 2 (GLP1, GLP2), pancreatic polypeptide (PP), and peptide YY (PYY)), and adipose tissue (leptin). Furthermore, in the hypothalamus, cannabinoids induce food intake via the cannabinoid receptor type 1 (CB1) [163, 164] (Figure 6).

**Figure 7:**
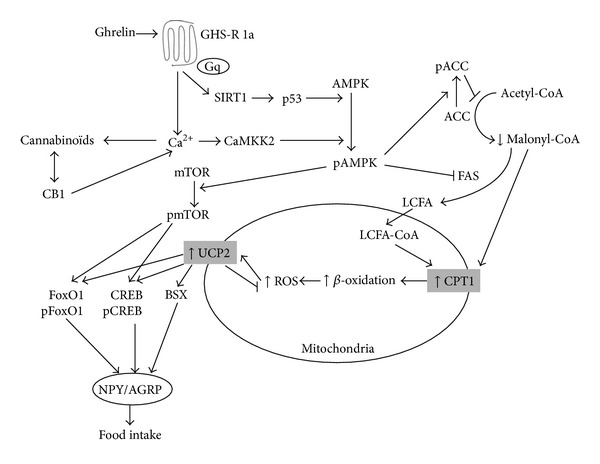
Molecular mechanisms leading to ghrelin-induced food intake in the hypothalamus. Intracellular signaling pathways and mitochondrial metabolism resulting in NPY/AGRP secretion consecutive to ghrelin receptor activation are schematized. ACC: acetyl coenzyme A carboxylase; AGRP: agouti-related protein; AMPK: 5′ adenosine monophosphate-activated protein kinase; BSX: brain-specific homeobox transcription factor; CaMKK2: calmodulin kinase-kinase 2; CB1: cannabinoid receptor type 1; CPT1: carnitine-palmitoyltransferase-1; CREB: cyclic adenosine 3′,5′ monophosphate response element-binding protein; FAS: fatty acid synthase; FoxO1: forkhead box protein O1; GHS-R1a: growth hormone secretagogue receptor type 1a; Gq: Gq protein; LCFA: long chain fatty acid; LCFA-CoA: long chain fatty acyl coenzyme A; mTOR: mammalian target of rapamycin; NPY: neuropeptides Y; p: phosphorylated state; ROS: reactive oxygen species; SIRT1: sirtuin 1; and UCP2: uncoupling protein-2.

Ghrelin stimulates appetite by central and peripheral pathways and via the vagus nerve. Indeed, ghrelin is locally synthesized in the hypothalamus [165], ghrelin secreted by the stomach reaches the brain by crossing the blood-brain barrier [166], and ghrelin also transmits its signal through the vagal nerve [167]. In hypothalamus, ghrelin activates the arcuate nucleus (ARC), paraventricular nucleus (PVN), dorsomedial region, central nucleus of amygdala, and the nucleus of solitary tract [168, 169]. Neurons expressing ghrelin send efferents to ARC neurons producing NPY, AGRP, POMC, and CRH [165]. By stimulating the activity of NPY/AGRP neurons and decreasing the activity of POMC and CART neurons, ghrelin increases appetite and food intake [165, 170, 171]. Ghrelin can directly inhibit PVN neurons [172] or activate NPY/AGRP neurons and inhibit POMC neurons that are in contact with PVN. Hypothalamic 5′ AMP-activated protein kinase (AMPK) has been proposed to play a pivotal role in ghrelin's effects on appetite and food intake [172, 173]. AMPK is a serine/threonine protein kinase sensing the energy status of the cells and regulates fuel availability by stimulating ATP producing pathways and inhibiting ATP consuming pathways [174]. Following ATP depletion, AMP rises and induces the activation of AMPK through its phosphorylation [175]. Activated AMPK induces the phosphorylation of acetyl-CoA carboxylase (ACC), leading to the inhibition of ACC activity and the decrease in malonyl-CoA levels and finally resulting in increased fatty acid oxidation via the activation of carnitine-palmitoyl transferase 1 (CPT1) [176, 177]. Following increased fatty acid *β*-oxidation, reactive oxygen species are generated and stimulate uncoupling protein-2 (UCP2) which promotes ROS scavenging and stimulates NPY/AGRP transcription [170]. The activation of the hypothalamic AMPK signaling cascade results in an increase of appetite and food intake (Figure 7).

Ghrelin has been shown to stimulate AMPK by phosphorylation via calmodulin kinase-kinase 2 (CaMKK2) activated in response to rise in intracellular calcium concentration induced by GHS-R1A signaling [165, 178, 179]. However, a recent study demonstrated that the effect of ghrelin on AMPK signaling pathway occurs independently from GHS-R1A, thereby suggesting that the AMPK signaling pathway does not play a major role in the orexigenic effect of ghrelin [180].

Ghrelin has also been shown to activate hypothalamic Sirtuin 1 (SIRT1)/p53 [181] and mammalian target of rapamycin (mTOR) [182, 183]. SIRT1 is a deacetylase activated in response to calorie restriction that acts through the tumor suppressor p53. SIRT1 and p53 are required for ghrelin-induced AMPK activation and consequent orexigenic action [181]. mTOR is a ser/threonine kinase acting as a cellular sensor of energy balance changes, growth factors, nutrients, and oxygen [184–188]. mTOR is regulated by the cellular AMP/ATP ratio; mTOR activity decreases when AMP/ATP increases (low energy), and conversely, mTOR activity increases when AMP/ATP ratio decreases (high energy) [189]. Hypothalamic mTOR signaling is involved in food intake [189, 190]. mTOR can be either inhibited [191, 192] or activated [193] by AMPK. In the arcuate nucleus of the hypothalamus, it appears that mTOR is activated by AMPK [193]. Activated mTOR phosphorylates S6-kinase-1 (S6K1), S6 ribosomal protein (S6), and initiation factor 4E-binding protein (4E-BP1) [184, 194]. In the hypothalamus, mTOR and S6K1 are only located on the NPY/AGRP and POMC neurons within the ARC. It has been shown that hypothalamic mTOR signaling mediates the orexigenic action of ghrelin [182, 193, 195]. Indeed, ghrelin-mediated mTOR activation induces the increase of CREB-pCREB, FoxO-pFoxO1, and BSX transcription factors which in turn activate NPY and AGRP synthesis, leading to food intake [182, 196] (Figure 7). These data appear to be opposed to those showing that activation of mTOR signaling promotes anorexia [190, 192].

Several studies have highlighted the relevance of cellular sensors, AMPK and mTOR, in the control of ghrelin-induced food intake. It is possible that the orexigenic effects of ghrelin mediated by AMPK, and mTOR occur in distinct part of the hypothalamus. Additional studies will be necessary to investigate this hypothesis, to further assess the interaction between AMPK and mTOR in response to ghrelin, and to better understand why ghrelin is able to activate both AMPK and mTOR signaling pathways.

The intact cannabinoids signaling pathway is required for the effect of ghrelin on appetite and AMPK [172, 197, 198]. Furthermore, control of AMPK signaling pathway by cannabinoids requires an intact ghrelin signaling pathway [199]. All together, these data suggest an interaction between GHS-R1A and CB1, possibly in the form of heterodimers as already shown for GHS-R1A and other GPCRs (see Section 4.2).

Both in GHSR and ghrelin knockout mice, food intake is similar to littermate wild-type mice [200, 201].

In light of the complexity of the hypothalamic ghrelin-signaling pathways, more studies are required to investigate more profoundly their relative importance in food intake and also investigate their possible activation in certain regions of the hypothalamus. Furthermore, additional studies will be required to better understand the interactions between ghrelin and cannabinoids signaling and their implication in the control of food intake.

Ghrelin also stimulates appetite via the vagus nerve. Human nodose ganglion from the vagus nerve expressing GHS-R1A are likely to be involved in the ghrelin-induced signal transmission from the stomach to the brain [202, 203]. Indeed, rats submitted to vagotomy or perivagal application of an afferent neurotoxin [140] or patients with vagotomy and esophageal or gastric surgery are responding to the appetite stimulatory effect of ghrelin [204, 205]. Thus, through the activation of GHS-R on vagal afferent to the stomach, the signal induced by ghrelin may reach the nucleus of tractus solitarius, which communicates with the hypothalamus to increase food intake. However, intraperitoneal injection of ghrelin stimulates eating in rats with subdiaphragmatic vagal deafferentation, suggesting that the ghrelin signal does not involve vagal afferents [206].

The clinical applications of ghrelin have been investigated in both eating disorders and muscle wasting conditions, including obesity, anorexia nervosa, cachexia, and sarcopenia (for a review see [151]).

### 5.3. Energy Homeostasis

Ghrelin is involved in long-term body weight regulation. Plasma ghrelin levels are negatively correlated with body weight in anorexia nervosa, cachexia, and obesity and fluctuate in a compensatory manner to body weight modifications [39]. Indeed, ghrelin levels decrease with weight gain resulting from different conditions such as overfeeding [207], pregnancy [208], olanzapine treatment [209], or high fat diet [210]. Conversely, ghrelin levels increase with weight loss resulting from conditions such as food restriction [211], long-term chronic exercise but not acute exercise [212], cachectic states induced by anorexia nervosa [39], severe congestive heart failure [213], lung cancer [214], and breast and colon cancers [215]. Data on ghrelin levels after weight loss induced by gastric bypass surgery remain controversial as some studies found a decrease [216–219], no change [220–222] or an increase of ghrelin levels [223–225]. However variations in surgical procedures and patient treatment may account for the discrepancies.

*In vivo*, chronic ghrelin administration induces adiposity [157, 226]. In addition to stimulate food intake, ghrelin reduces energy expenditure, consequently decreasing utilization and oxidation of fat while increasing utilization of carbohydrates [227]. *In vitro*, ghrelin stimulates differentiation of preadipocytes [129], adipogenesis [228], inhibits adipocyte apoptosis [229], and antagonizes lipolysis [230, 231]. Furthermore, ghrelin shifts food preference towards high fat diets [232].

GHSR and ghrelin knockout mice have been useful in determining the role of ghrelin in energy homeostasis. GHSR knockout mice, with C56Bl6J background, fed on normal diet displayed slight decrease in body weight, with no modification of food intake, as compared to wild-type mice [200]. However, GHSR knockout mice, with C56Bl6J:129sv background, fed on normal diet displayed normal body weight, but exhibited resistance to diet-induced obesity and lower fat mass when fed on high fat diet, compared to wild-type mice [233]. These latter observations are likely to result from the effect of the genetic background as C56Bl6J:129sv mice are inherently more resistant to diet-induced obesity compared to C56Bl6J knockout mice. Ghrelin knockout mice displayed normal food intake, body weight, and body composition when compared to wild-type littermates [201, 234]. Furthermore, ghrelin knockout mice presented intact hypothalamic regulatory feeding centers [234]. Ghrelin knockout mice fed with high fat diet were resistant to diet-induced obesity [201, 227, 234]. Very recently, it was shown that congenic adult ghrelin knockout mice submitted to either a positive (high-fat diet) or negative (caloric restriction) energy balance displayed similar body weight as wild-type littermates [235]. These contradictory data could be explained by differences in the mouse genetic backgrounds and/or the moment at which the high-fat diet was given to the animals. However, interpretation of ghrelin knockout mice should be taken with caution as proghrelin yields several other peptides besides ghrelin that may play roles in the overall metabolism linked to ghrelin itself. Further studies are required to determine if ghrelin plays a role in the development of obesity.

In patients with Prader-Willi syndrome (PWS), a genetic disorder characterized by mental retardation and hyperphagia leading to severe obesity, plasma ghrelin levels are higher than in healthy subjects and do not decrease after a meal [236, 237]. Other studies showed that ghrelin levels decreased postprandially in adult patients with PWS, but to a lesser extent than in obese and lean subjects [238, 239]. This lesser postprandial ghrelin suppression may be due to a blunted postprandial response of PYY, an anorexigenic peptide that decreases postprandial ghrelin levels. The low PYY levels could partially explain the high ghrelin levels observed in PWS [238]. Interestingly, children (5 years of age and younger) with PWS have normal ghrelin levels. Since these children have not yet developed hyperphagia or excessive obesity; it suggests that ghrelin levels increase with the onset of hyperphagia [240, 241]. In opposition with these data, plasma ghrelin levels in children with PWS were elevated at any age, including the first years of life, thus preceding the development of obesity [242]. Thus, ghrelin may be responsible, at least partially, for the insatiable appetite and the obesity of these patients.

In the liver, ghrelin inhibits AMPK activity [164] and increases the expression and activity of key enzymes involved in fat metabolism such as stearoyl-CoA desaturase 1 (SCD1), acetyl CoA carboxylase (ACC), and fatty acid synthase (FAS) [243, 244]. In adipose tissue, ghrelin also inhibits AMPK activity and increases the expression of peroxisome proliferators-activated receptor *γ* (PPAR*γ*) and sterol-regulatory element binding protein-1 (SREBP1) (two transcription factors involved in promoting adipogenesis) as well as ACC, FAS, lipoprotein lipase, and perilipin [231, 244]. These peripheral actions of ghrelin require p53 [245] and cannabinoid receptor type 1 (CB1) [198].

### 5.4. Gastric Secretion and Gastrointestinal Motility

Ghrelin, administrated peripherally, dose-dependently increases gastric acid secretion [246, 247], by a mechanism involving the vagus nerve [140, 247] and histamine synthesis and release [248]. Furthermore, ghrelin acts in synergy with gastrin to stimulate gastric acid secretion [249, 250]. Ghrelin, administrated centrally, either induces [251] or inhibits [252, 253] gastric acid secretion and possibly involves the vagus nerve. Furthermore, gastric acid secretion induced by ghrelin involves nitric oxide pathway [254, 255].

*In vitro*, ghrelin dose-dependently enhances the after-contraction of gastric smooth muscle cells elicited during electrical field stimulation [256–258]. Furthermore, ghrelin acts on cholinergic and tachykinergic neurotransmission [257–259]. Ghrelin has no effect *in vitro* on the contractility of human and rodent colon muscle strips [256, 260–262].

*In vivo*, several studies have shown a dose-dependent effect of ghrelin on gastric emptying and intestinal transit following peripheral or central administration of ghrelin in rodents [247, 257, 263–267]. The vagus nerve is involved in the prokinetic action of ghrelin [246, 247, 263].

Ghrelin also activates the migrating motor complex in rodent stomach and small intestine [268–270] by a mechanism involving the vagus nerve [268]. However, a contradictory study showed no effect of ghrelin on the migrating motor complex in mice [271].

In agreement with *in vitro* studies on colonic contractility [256, 261], peripheral administration of ghrelin has no effect on colonic transit in rodents [266, 272]. However, central administration of ghrelin stimulates colonic motility [273, 274]. The poor capacity of ghrelin to cross the blood-brain barrier could account for the lack of peripheral administration of ghrelin on colonic motility.

In humans, peripheral administration of ghrelin induces accelerated gastric emptying [275] with no modification of orocecal and colonic transit [276]. Besides, ghrelin stimulates the human migrating motor complex [277].

Furthermore, ghrelin has been shown to have a series of important therapeutic potentials for the treatment of gastrointestinal motility disorders [151, 255]. Therefore, future studies are needed to study the beneficial effects of novel ghrelin receptor agonists in gastrointestinal motility disorders.

### 5.5. Glucose Homeostasis

Numerous studies support a role for ghrelin in blood glucose homeostasis [130, 278, 279]. Glucose homeostasis is regulated by insulin secreted by pancreatic *β* cells and the insulin responsiveness of peripheral tissues. Both the effects of ghrelin on insulin secretion and vice versa have been described. The inverse relationship between blood ghrelin levels and insulin levels has suggested the existence of an inhibitory feedback between ghrelin and insulin [8, 280, 281]. Ghrelin was first demonstrated to negatively affect insulin secretion in human [282]. However, depending on experimental conditions, ghrelin either stimulates [283, 284] or inhibits insulin secretion [282, 285]. Indeed, ghrelin might have an inhibitory effect on insulin secretion at low concentrations and a stimulatory effect at high concentration [286]. The mechanisms involved in the inhibitory effect of ghrelin on glucose-induced insulin secretion include an increase expression of the insulinoma-associated protein 2*β* (IA-2*β*) [287] and the activation of the AMPK-uncoupling protein 2 (UCP2) pathway [288, 289].

In human, ghrelin was shown to decrease insulin sensitivity depending on the acyl ghrelin/desacyl ghrelin ratio as acyl ghrelin promotes insulin desensitization, while desacyl ghrelin neutralizes the insulin desensitizing effects of acyl ghrelin [132, 290, 291].

Insulin decreases ghrelin levels independently of glucose concentrations [278, 292–294]. However, contradictory data showed no negative effect of insulin on ghrelin concentrations [295, 296]. These discrepancies could be due to different experimental settings.

Maintenance of glucose homeostasis includes the ability of the central nervous system to sense changes in glucose levels. Increased ghrelin levels during preprandial and fasting periods [161, 297, 298] and hypothalamic GHSR expression [299] suggest the involvement of a central mechanism whereby the ghrelin system can sense decreasing glucose concentrations [300]. Indeed, AGRP and NPY expressing neurons from the hypothalamic arcuate nucleus are modulated by glucose [301]. Besides, glucose responding neurons are also present in hypothalamic ventromedial nucleus, lateral hypothalamic area, and the parvocellular area of the paraventricular nucleus. These regions represent important targets for the orexigenic and energy homeostatic effects of ghrelin [165, 302], and glucose sensing neurons respond to ghrelin [303]. Moreover, GHSR is expressed adjacent to these hypothalamic regions [303, 304]. Activation of neurons from the nucleus of solitary tract by insulin-induced hypoglycemia triggers an orexigenic response involving neurons containing ghrelin [305, 306]. Future studies are warranted to determine how ghrelin leads to insulin counter-regulatory effects and central control of glucose homeostasis.

Ghrelin stimulates liver glycogenolysis and neoglucogenesis and prevents suppression of glucose production by insulin, and thereby participate to increased blood glucose concentrations [307–310]. However, desacyl ghrelin dose-dependently inhibits liver glucose production [307]. A better understanding of the modulation of hepatic glucose production by acyl ghrelin/desacyl ghrelin ratio, and its influence on glucose homeostasis, requires additional studies, especially to unravel the mechanism of action of desacyl ghrelin.

GOAT could be involved in glucose homeostasis as well. Indeed GOAT knockout mice present improved glucose-induced insulin secretion and glucose tolerance [41]. Besides, GOAT knockout mice submitted to severe caloric restriction displayed severe hypoglycemia that can result into death [41]. However, those data have not been confirmed in another study using GOAT knockout mice submitted to caloric restriction [311]. Distinct genetic background of the mouse strains uses and experimental conditions could account for these discrepancies. It remains to be proven, by future studies, if GOAT indeed plays a role in the control of glucose homeostasis.

In summary, the role of ghrelin and GOAT in the control of glucose homeostasis remains controversial and mechanistically poorly understood. However, due to the crucial role of glucose homeostasis, certainly further studies are required to address these issues. Nevertheless, due to the possible involvement of ghrelin in the control of glucose homeostasis, ghrelin receptor already represents a therapeutic target for the treatment of type 1 and type 2 diabetes [151, 279].

### 5.6. Cardiovascular Functions

Ghrelin has been shown to have diverse cardiovascular functions [312–314]. Ghrelin decreases mean arterial pressure without altering the heart rate in healthy subjects [315]. In animal models with heart failure, ghrelin improved cardiac output and contractility and attenuated left ventricular remodeling and development of cachexia [316]. In patients with chronic heart failure, ghrelin improved left ventricular function, increased cardiac output and cardiac index, decreased systemic vascular resistance, and increased muscle strength [317]. Ghrelin improved cardiac contractility and left ventricular function in chronic heart failure and reduced infarct size [318]. The mechanisms responsible for the hypotensive effects of ghrelin include the suppression of sympathetic activity [319] or direct vasodilatory action [320–322]. Ghrelin dilates human artery in an endothelium-independent manner [320, 323]. In rats with myocardial infarction, chronic ghrelin treatment suppressed cardiac sympathetic activity and prevented early left ventricular remodeling [324], while acute ghrelin treatment improved survival by preventing the increase of frequency of ventricular arrhythmias [325]. Furthermore, the vasodilatory action of ghrelin involves activation of PI3 kinase, AKT, and endothelial nitric oxide synthase [321, 322]. Interestingly, desacyl ghrelin is as potent as acyl ghrelin in exerting cardioprotective effects, probably by acting on an as yet unidentified receptor distinct from GHS-R1A [326]. Besides, desacyl ghrelin improves vascular neovascularisation and, namely, in diabetic patients. Ghrelin is able to block the rennin-angiotensin system in humans and thereby improve hypertension and cardiovascular disorders as well as conditions associated with increased risk of developing cardiovascular disease such as disorders of glucose metabolism, dyslipidemia, and inflammatory states [327]. Ghrelin protects the heart against ischemia/reperfusion injury [328].

Ghrelin inhibits apoptosis of cardiomyocytes and endothelial cells possibly through the activation of extracellular signal-regulated kinase 1/2 and Akt serine kinases [124].

Ghrelin plays multiple beneficial cardiovascular functions, thereby improving cardiovascular disease. Additional investigations are required for a thorough understanding of the detailed functions of ghrelin in the cardiovascular system in normal and pathological conditions. Surely, the usefulness of ghrelin analogs for the treatment of cardiovascular disease remains to be fully addressed and proven by adequate clinical studies.

### 5.7. Anti-Inflammatory Functions

Several studies have reported that ghrelin is able to exert anti-inflammatory actions by inhibiting the production of proinflammatory cytokines [329–332]. Indeed, ghrelin exerts anti-inflammatory actions in inflammatory bowel disease, pancreatitis, sepsis, arthritis, and diabetic nephropathy [333–341].

Despite limited clinical value, ghrelin administration prior to the development of experimental pancreatitis improved pancreatic blood flow, reduced IL1*β* levels, and stimulated pancreatic cell proliferation [333]. In sepsis, ghrelin, via an upregulation of MAPK phosphatase 1, reduced norepinephrine and TNF*α* levels known to cause hepatocellular dysfunction and upregulation of proinflammatory cytokines [342]. Furthermore, organ blood flow is improved by ghrelin via an inhibition of NF-kB [343] and HMGB1 production by activated macrophages is inhibited by ghrelin [335]. Ghrelin reduced IL6 levels and symptoms of arthritis in an animal model [334]. IL6 and IL8 levels induced by insoluble fibrillary *β*-amyloid protein deposition in mouse microglia are decreased by desacyl ghrelin but not by acyl ghrelin probably by a mechanism involving, as already eluded to, an unidentified receptor distinct from GHS-R1A [344]. Antihyperalgesic and anti-inflammatory effects of both acyl ghrelin and desacyl ghrelin have been shown in rats [340]. Development of experimental diabetic nephropathy in mice can be prevented by acyl ghrelin acting on GHS-R1A [341]. Inflammatory bowel disease, in particular Crohn's disease, was improved by ghrelin administration [336].

Further studies are definitively required to evaluate the potential benefit of ghrelin treatment for inflammatory-related conditions.

### 5.8. Reproductive Functions

Ghrelin controls several aspects of female and male reproductive physiology and pathology through endocrine and autocrine/paracrine pathways [345–349]. Ghrelin affects the hypothalamic-pituitary gonadal axis as well as female and male reproduction systems.

Systemic actions of ghrelin on the hypothalamic-pituitary gonadal axis include inhibition of hypothalamic gonadotropin-releasing hormone (GnRH) and of both LH and FSH secretion [350–352] and stimulation of prolactin [353]. Identification of the precise mechanisms accounting for the effects of ghrelin on the hypothalamic-pituitary gonadal axis will require further experimentation.

In the female reproduction system, both ghrelin and GHS-R are present in ovary [354–358]. Depending on species, ghrelin exerts an inhibitory or stimulatory effect on steroidogenesis (progesterone and estradiol production) [359–361]. Ghrelin promotes proliferation and inhibits apoptosis of ovarian cells [358, 362]. Both ghrelin and GHS-R have been detected in oocytes and different stages of embryo development [363]. The effects of ghrelin on embryo development remain controversial [347, 348]. Both ghrelin and GHS-R have been detected in placenta from several species [364, 365]. Ghrelin stimulates the proliferation, inhibits the apoptosis, decreased progesterone secretion, and did not modify human chorionic gonadotropin (hCG) secretion of human placental JEG-3 cells [366].

In the male reproduction system, both ghrelin and GHS-R are localized in testis, mainly in Leydig and Sertoli cells, but their localization varies depending on species [347–349]. Ghrelin regulates testicular stem cell factor and impairs Leydig cell proliferation [367]. Ghrelin also inhibits hCG- and cAMP-stimulated testosterone release by Leydig cells [368–370]. It has been suggested that elevated ghrelin levels could contribute to male reproductive alterations, especially in situations of energy deficiency [371].

Further studies are required to gain insights into the understanding of the detailed mechanism of action of ghrelin in the female and male reproductive systems.

### 5.9. Bone Formation

Bone formation is induced by ghrelin that stimulates osteoblastic cell proliferation and differentiation, inhibits cell apoptosis, and increases bone mineral density [372–375].

In several species, ghrelin stimulates osteoblast proliferation and differentiation but inhibits apoptosis [376, 377]. PI3K and MAPK pathways are likely to be involved in the ghrelin-induced proliferation of osteoblasts [378]. The effects of ghrelin on osteoblast could either result from endocrine [379] or autocrine/paracrine effects [374, 378]. The absence of GHS-R1A expression and the presence of GHS-R1b expression [13, 378] on osteoblasts suggest that ghrelin-mediated effects are mediated by an as yet unidentified mechanism.

The role of ghrelin on osteoclast function remains poorly understood as it has been shown to either enhance osteoclast resorption [380] or inhibit osteoclast differentiation [381].

Ghrelin modulates chondrocyte function likely by an autocrine/paracrine pathway independent of GHS-R1A [382].

Chronic central ghrelin administration increases rat bone mass through a mechanism independent of appetite regulation [383]. Per os ghrelin administration induces new bone formation and stimulates intramembranous bone repair of calvarial bone defects in rats [384]. In human, blood ghrelin level was positively correlated with bone mineral density in perimenopausal, postmenopausal, and premenopausal women, while it was significantly decreased in perimenopausal and postmenopausal women as compared to premenopausal women [385]. In elderly women, but not in men, ghrelin levels were associated with trabecular bone mass density but not with total or cortical bone mass density [386]. In obese adolescent girls, ghrelin is a negative predictor for bone mineral density and content [387]. In a randomised, double-blind, placebo-controlled study, ghrelin infusion had no acute effect on markers of bone turnover in healthy controls and postgastrectomy subjects but was inversely correlated with bone resorption [388].

Further studies are needed to precise the molecular mechanisms involved in ghrelin-mediated effects on the different bone cell types and on bone formation, and to investigate its potential use to treat elderly patients suffering from osteoporosis or at risk.

## 6. Conclusions 

Ghrelin is peptide hormone that is essentially secreted by the stomach into the blood stream, but other tissues have been shown to also synthesize it. Ghrelin can exert its effects through systemic or autocrine/paracrine actions. GHS-R1A receptor binds acyl ghrelin and is supposed to mediate its biological effects. However, it is recognized that both GHS-R1A homo- or heterodimers could be involved in the ghrelin-mediated actions. The formation of homo- and heterodimers is adding another level of complexity in the understanding of the actions of ghrelin. Growing bodies of evidence support an increased number of functions for desacyl ghrelin. However the exact mechanisms and a potential specific receptor have thus far eluded determination. Much work remains to be done to determine if this additional level of complexity is indeed accounting for the biological effects of ghrelin. Numerous and varied physiological effects of ghrelin, as reviewed in this paper, have been reported. However, it appears important to perform further studies to better understand the fine underlying mechanisms accounting for these pleiotropic ghrelin actions. Furthermore, current understanding of ghrelin biology and biological functions has led to the development of pharmacological tools modulating ghrelin actions and the evaluation of their clinical applications.
